# In-Hospital Mortality of Cardiogenic Shock After Acute Myocardial Infarction: A Prospective Study

**DOI:** 10.7759/cureus.112875

**Published:** 2026-07-17

**Authors:** Shahab Shahab, Ubaidullah Popal

**Affiliations:** 1 Department of Cardiology, Ayub Teaching Hospital, Abbottabad, PAK; 2 Department of Cardiology, Midlands Metropolitan University Hospital, Birmingham, GBR; 3 Department of Cardiology, Tabba Heart Institute, Karachi, PAK

**Keywords:** cardiogenic shock, in-hospital mortality, myocardial infarction, risk factors, stemi

## Abstract

Background and objective

Cardiogenic shock (CS) following acute ST-segment elevation myocardial infarction (STEMI) is a life-threatening complication associated with high in-hospital mortality despite advances in reperfusion and critical care. Published evidence from South Asia remains limited, and the independent contribution of time-dependent treatment variables to mortality in this setting is poorly characterized. The objective of this study is to determine the frequency of in-hospital mortality and its independent predictors among patients with CS following acute STEMI.

Methodology

The study was conducted as a prospective observational cohort study in the Department of Cardiology, Ayub Teaching Hospital, Abbottabad, Pakistan, from April to December 2021. Using consecutive non-probability sampling, 135 patients aged 18-80 years with STEMI and CS were enrolled. A structured proforma was used to gather data on demographic characteristics, comorbidities, reperfusion strategy (primary percutaneous coronary intervention (PPCI), thrombolysis, or conservative medical management), symptom-to-door time, total ischemic time, door-to-balloon time, door-to-needle time, and clinical outcomes. In-hospital mortality was evaluated during the hospital stay. Data were analyzed with IBM SPSS Statistics for Windows, Version 20 (Released 2011; IBM Corp., Armonk, NY, USA). Univariate analysis (chi-square test, Mann-Whitney U test, and logistic regression) was performed to identify associations, and multivariate logistic regression with backward elimination was used to determine independent predictors. A two-sided p-value < 0.05 was considered significant.

Results

Among 135 patients, the mean age was 55.03 ± 13.06 years, and 68 (50.37%) were male. Diabetes mellitus was present in 81 (60.00%), hypertension in 94 (69.63%), and smoking in 28 (20.74%) patients. PPCI was performed in 80 (59.26%), thrombolytic therapy in 31 (22.96%), and medical therapy only in 24 (17.78%) patients. The median symptom-to-door time was 3.70 hours (IQR 2.63-5.33), and the median total ischemic time was 5.10 hours (IQR 3.86-6.83). In-hospital mortality occurred in 48 patients (35.56%). Multivariate analysis identified four independent predictors of mortality: diabetes mellitus (adjusted OR 37.67, 95% CI 4.94-287.29, p < 0.001), hypertension (adjusted OR 170.50, 95% CI 5.72-5081.40, p = 0.003), anterior wall MI (adjusted OR 27.06, 95% CI 5.17-141.74, p < 0.001), and symptom-to-door time (adjusted OR 1.89 per hour, 95% CI 1.19-2.99, p = 0.007). The crude protective association of smoking (OR 0.05) was fully attenuated after adjustment (aOR 0.19, p = 0.234), consistent with the smoker's paradox.

Conclusion

CS in STEMI patients is associated with high in-hospital mortality. Diabetes, hypertension, anterior infarct location, and prolonged symptom-to-door time are independent predictors of death. Every hour of pre-hospital delay increases mortality risk by 89% after controlling for comorbidities and reperfusion strategy, underscoring the critical importance of rapid hospital presentation in this high-risk population.

## Introduction

Acute ST-segment elevation myocardial infarction (STEMI) remains one of the leading causes of cardiovascular morbidity and mortality worldwide despite major advances in pharmacological therapy and timely reperfusion strategies. Rapid coronary occlusion results in prolonged myocardial ischemia and irreversible myocardial necrosis if coronary blood flow is not promptly restored, thereby increasing the risk of life-threatening complications and adverse clinical outcomes. Although primary percutaneous coronary intervention (PPCI) has substantially improved survival among patients with STEMI, mortality remains considerable in those who develop hemodynamic complications during the acute phase of infarction [1-4].

Cardiogenic shock (CS) is the most severe complication of acute STEMI and is characterized by persistent hypotension secondary to profound cardiac pump failure, leading to systemic hypoperfusion, end-organ dysfunction, and multiorgan failure. Contemporary STEMI registries indicate that CS develops in approximately 5%-10% of patients with acute myocardial infarction (AMI), yet it accounts for a disproportionately large share of early deaths. Despite advances in reperfusion therapy and intensive cardiac care, reported in-hospital mortality remains approximately 35%-50% in patients receiving contemporary treatment and may exceed 60% in high-risk populations or delayed presentations. Long-term outcomes also remain poor, with one-year mortality approaching 45%-60% among survivors of the acute episode. These findings underscore CS as the principal determinant of mortality following acute STEMI [5-9].

Several demographic and clinical characteristics have been associated with adverse outcomes in STEMI complicated by CS, including advanced age, diabetes mellitus, hypertension, extensive anterior wall infarction, delayed reperfusion, impaired left ventricular function, and multivessel coronary artery disease. Although large randomized trials and multicenter registries have identified these prognostic factors, their relative contribution varies across healthcare systems because patient characteristics, treatment pathways, availability of specialist cardiac services, and critical care resources differ substantially between high-income and resource-constrained settings [10-12].

In Pakistan, STEMI continues to represent a major burden on tertiary cardiac care services. However, timely access to PPCI remains inconsistent; many patients present after prolonged ischemic time, and advanced cardiac critical care resources, including dedicated shock teams and mechanical circulatory support devices such as intra-aortic balloon pumps, Impella, or extracorporeal membrane oxygenation (ECMO), are not universally available. Consequently, the clinical profile and outcomes of patients with STEMI complicated by CS in Pakistani tertiary-care hospitals may differ from those reported in large international registries. Nevertheless, contemporary local data describing in-hospital mortality and its associated clinical characteristics remain limited, particularly from northwestern Pakistan, where tertiary referral centers manage a substantial proportion of critically ill cardiovascular patients.

Therefore, the present prospective observational cohort study was conducted at Ayub Teaching Hospital, Abbottabad, Pakistan, to address this important evidence gap. The primary objective was to determine the frequency of in-hospital mortality among patients presenting with CS following acute STEMI. The secondary exploratory objective was to evaluate the association between selected baseline demographic and clinical characteristics and in-hospital mortality, thereby providing locally relevant evidence to support risk stratification, optimize clinical decision-making, and guide future quality improvement initiatives in resource-limited settings.

## Materials and methods

Study design and setting

This prospective observational cohort study was conducted in the Department of Cardiology, Ayub Teaching Hospital, Abbottabad, Pakistan, from April to December 2021. Ayub Teaching Hospital is a tertiary-care teaching and referral hospital serving Abbottabad and the surrounding districts of Khyber Pakhtunkhwa. The Department of Cardiology receives referrals of patients with acute coronary syndromes from both secondary and primary healthcare facilities across the region and provides round-the-clock emergency cardiac care through its Coronary Care Unit (CCU).

During the study period, patients with suspected STEMI were managed according to institutional protocols based on contemporary guideline-directed management. The standard reperfusion strategy consisted of PPCI whenever available within the recommended time window; otherwise, fibrinolytic therapy was administered when PPCI was not feasible or timely. Patients with CS received intensive hemodynamic monitoring, vasoactive medications, ventilatory support when indicated, and other supportive measures in the CCU. The department was staffed by experienced consultant cardiologists, senior registrars, resident medical officers, and house officers, providing continuous 24-hour coverage. The hospital was equipped with facilities for routine and specialized cardiovascular investigations and functioned as a major referral center for the management of acute cardiovascular emergencies in the region.

Sampling size and sampling technique

The sample size was calculated using the WHO sample size calculator based on an anticipated in-hospital mortality of 34.1% among patients with CS complicating acute STEMI, as reported by Venkatason et al. [13] in a retrospective analysis of the Malaysian National Cardiovascular Database (NCVD) registry. Assuming a 95% CI, an absolute precision (margin of error) of 8%, and an expected mortality proportion of 34.1%, the required sample size was calculated to be 135 patients. Eligible participants were enrolled consecutively using a non-probability consecutive sampling technique until the required sample size was achieved.

Study population

The study comprised adult patients aged 18-80 years of either sex who were admitted with acute STEMI complicated by CS. CS was defined by persistent systolic blood pressure <90 mmHg (or the requirement for vasopressor/inotropic support to maintain systolic blood pressure ≥90 mmHg) for at least 30 minutes, accompanied by clinical evidence of tissue hypoperfusion, including cold extremities, altered mental status, oliguria, or elevated serum lactate, in the absence of hypovolemia. Only patients with CS attributable to acute left ventricular pump failure secondary to STEMI were included. Patients with CS resulting from mechanical complications of myocardial infarction (including ventricular septal rupture, papillary muscle rupture with acute severe mitral regurgitation, or free-wall rupture), non-STEMI (NSTEMI), hypotension due to non-cardiac causes (e.g., septic or anaphylactic shock), or STEMI without CS were excluded to minimize clinical heterogeneity and potential confounding.

Development and implementation of the structured CS-STEMI questionnaire

Following approval from the Institutional Ethics Committee, consecutive eligible patients admitted to the Department of Cardiology, Ayub Teaching Hospital, Abbottabad, Pakistan, with acute STEMI complicated by CS were enrolled. As many patients were critically ill at presentation, written informed consent was obtained from the patient's legally authorized representative or next of kin.

For this study, the investigators designed and developed a structured CS-STEMI Questionnaire to ensure standardized and comprehensive data collection (Appendix 1). The questionnaire was developed based on the study objectives, current STEMI management guidelines, and relevant published literature and was used prospectively for all enrolled patients. It included sections on demographic characteristics, clinical presentation, cardiovascular risk factors, medical history, physical examination findings, laboratory and electrocardiographic findings, treatment details, and in-hospital outcomes.

Using the structured CS-STEMI Questionnaire, baseline demographic and clinical information, physical examination findings, and relevant comorbidities, including diabetes mellitus, hypertension, smoking status, and body mass index (BMI), were systematically documented. Data regarding reperfusion strategy (PPCI, thrombolytic therapy, or conservative medical management without reperfusion), symptom onset time, hospital arrival time, reperfusion time (balloon inflation for PCI or thrombolytic administration), and door-to-balloon or door-to-needle times were obtained from emergency department triage records, catheterization laboratory procedural logs, and medication administration records. Time-dependent variables, including symptom-to-door time and total ischemic time (hours), as well as door-to-balloon and door-to-needle times (minutes), were calculated and independently verified by two investigators to ensure data accuracy and consistency.

Patients received guideline-directed medical therapy according to institutional protocols, including antiplatelet agents, anticoagulation, vasopressor and/or inotropic support, intensive care monitoring, and reperfusion therapy, where indicated. The primary outcome was in-hospital mortality, which was assessed daily from the time of CCU admission until hospital discharge or death. Consecutive patient enrollment and the application of predefined inclusion and exclusion criteria minimized selection bias, while standardized data collection using the investigator-designed CS-STEMI Questionnaire enhanced the completeness, consistency, and reproducibility of the study data.

Statistical analysis

Data were entered and analyzed using IBM SPSS Statistics for Windows, Version 20 (Released 2011; IBM Corp., Armonk, NY, USA). Continuous variables, including age, BMI, symptom-to-door time, total ischemic time, door-to-balloon time, and door-to-needle time, were assessed for normality using the Shapiro-Wilk test and are presented as mean ± standard deviation (SD) or median (interquartile range, IQR), depending on the distribution. Categorical variables are presented as frequencies and percentages. The primary outcome was in-hospital mortality, which is reported as a proportion with its 95% confidence interval (CI). Associations between baseline demographic and clinical characteristics and in-hospital mortality were explored using the chi-square test or Fisher's exact test, where appropriate, for categorical variables. For continuous variables with non-normal distributions, the Mann-Whitney U test was used to compare groups. Crude ORs with corresponding 95% CIs were calculated to quantify the strength of association for categorical variables; for continuous variables, univariate logistic regression was performed, and the OR per unit increase (per hour for time variables) was reported. A two-sided p-value < 0.05 was considered statistically significant. No missing data were observed for the variables included in the analysis; therefore, complete-case analysis was performed. Multivariable logistic regression analysis with backward elimination (likelihood ratio) was subsequently performed to identify independent predictors of in-hospital mortality. Variables with p < 0.05 in univariate analysis, along with clinically relevant variables (age, gender, reperfusion strategy), were entered into the full model. Non-significant variables (p > 0.05) were sequentially removed, and the final model was selected based on the lowest Akaike Information Criterion (AIC). The Hosmer-Lemeshow goodness-of-fit test was used to assess model calibration, and the variance inflation factor (VIF) was checked to rule out multicollinearity (VIF < 5 for all retained variables). Adjusted ORs (aORs) with 95% CIs were calculated for the final model. The guideline of at least 10 outcome events per predictor variable was followed to avoid overfitting.

Ethical considerations

The study was conducted after obtaining approval from the CPSP. Written informed consent was obtained from the patients' legally authorized representatives before enrollment. Confidentiality and anonymity of all participants were maintained by assigning unique identification codes and securely storing the collected data.

## Results

The study included 135 patients with a mean age of 55.03 ± 13.06 years (range: 18-80) and an almost equal gender distribution, with 68 (50.37%) males and 67 (49.63%) females (Table 1). The mean BMI was 27.41 ± 3.51 kg/m² (range: 22-31), with 28 (20.74%) having normal weight, 68 (50.37%) overweight, and 39 (28.89%) obese. Diabetes mellitus was present in 81 (60.00%) patients, while 94 (69.63%) had hypertension and 28 (20.74%) were smokers. Regarding STEMI types, acute anterior wall MI was observed in 41 (30.37%), lateral wall MI in 54 (40.00%), and inferior wall MI in 40 (29.63%) patients. In-hospital mortality occurred in 48 (35.56%) patients, while 87 (64.44%) survived.

**Table 1:** Baseline Demographic, Clinical, Reperfusion, and Time Characteristics of STEMI Patients with Cardiogenic Shock (n = 135) Data are presented as n (%) or mean ± standard deviation (SD). ¹For primary PCI patients; ²For thrombolysis patients. STEMI: ST-segment elevation myocardial infarction; MI: myocardial infarction; PCI: percutaneous coronary intervention; IQR: interquartile range

Category	Variable	Value
Demographic Characteristics	Age (years), Mean ± SD	55.03 ± 13.06
	Male	68 (50.37)
	Female	67 (49.63)
Anthropometric Characteristics	BMI (kg/m²), Mean ± SD	27.41 ± 3.51
	Normal weight	28 (20.74)
	Overweight	68 (50.37)
	Obese	39 (28.89)
Clinical Characteristics	Diabetes mellitus, Yes	81 (60.00)
	Diabetes mellitus, No	54 (40.00)
	Hypertension, Yes	94 (69.63)
	Hypertension, No	41 (30.37)
	Smoking status, Smoker	28 (20.74)
	Smoking status, Non-smoker	107 (79.26)
Infarct Characteristics	Anterior wall MI	41 (30.37)
	Lateral wall MI	47 (34.81)
	Inferior wall MI	47 (34.81)
Reperfusion Strategy	Primary PCI	80 (59.26)
	Thrombolytic therapy	31 (22.96)
	Medical therapy only (no reperfusion)	24 (17.78)
Time Parameters	Symptom onset to hospital arrival (hours), Median (IQR)	3.70 (2.63-5.33)
	Total ischemic time (hours), Median (IQR)	5.10 (3.86-6.83)
	Door-to-balloon time (min), Median (IQR)¹	94.1 (72.7-114.5)
	Door-to-needle time (min), Median (IQR)²	30.0 (27.1-41.1)
Clinical Outcome	Died	48 (35.56)
	Survived	87 (64.44)

Table 2 shows the association of demographic and clinical characteristics with in-hospital mortality among 135 STEMI patients. Mortality was numerically higher among females than males (40.30% vs. 30.88%); however, this difference did not reach statistical significance (p = 0.253). Likewise, patients aged 41-80 years demonstrated a higher mortality rate than those aged 18-40 years (38.46% vs. 16.67%), representing a trend toward increased mortality that did not achieve statistical significance (p = 0.072). Mortality did not differ significantly across BMI categories (p = 0.594). In contrast, diabetes mellitus was significantly associated with increased in-hospital mortality, with 45 (55.56%) deaths among diabetic patients compared with three (5.56%) among non-diabetic patients (p < 0.001). Hypertension was also significantly associated with mortality, with 47 (50.00%) deaths among hypertensive patients versus one (2.44%) among non-hypertensive patients (p < 0.001). Smoking status demonstrated a statistically significant inverse association with mortality, with one (3.57%) death among smokers compared with 47 (43.93%) deaths among non-smokers (p < 0.001). Given the observational nature of the study and the absence of multivariable adjustment, this finding should be interpreted cautiously and may reflect residual confounding, differences in baseline characteristics, or the so-called "smoker's paradox" rather than a true protective effect of smoking. STEMI location was strongly associated with in-hospital mortality (p < 0.001), with the highest mortality observed among patients with anterior wall myocardial infarction (82.93%), followed by inferior wall (27.50%) and lateral wall myocardial infarction (5.56%).

**Table 2:** Association of Demographic and Clinical Characteristics with In-Hospital Mortality Among STEMI Patients (n = 135) Data are presented as n (%) for categorical variables and mean ± standard deviation for continuous variables. Categorical variables were compared using the χ² test; continuous variables were compared using the Mann-Whitney U test and analyzed using univariate logistic regression. Crude odds ratios (ORs) represent unadjusted associations. *A p-value < 0.05 was considered statistically significant; † denotes the Mann-Whitney U statistic.

Variable	Category	Died n (%)	Survived n (%)	Statistical Test Value	p-value	Crude OR (95% CI)
Gender	Female	27 (56.25)	40 (45.98)	1.31	0.253	Reference
	Male	21 (43.75)	47 (54.02)			0.66 (0.33-1.35)
Age group (years)	18-40	3 (6.25)	15 (17.24)	3.23	0.072	Reference
	41-80	45 (93.75)	72 (82.76)			3.12 (0.86-11.40)
Diabetes mellitus	No	3 (6.25)	51 (58.62)	35.35	<0.001*	Reference
	Yes	45 (93.75)	36 (41.38)			21.25 (6.12-73.74)
Hypertension	No	1 (2.08)	40 (45.98)	28.18	<0.001*	Reference
	Yes	47 (97.92)	47 (54.02)			40.00 (5.28-303.08)
Smoking status	Non-smoker	47 (97.92)	60 (68.97)	15.77	<0.001*	Reference
	Smoker	1 (2.08)	27 (31.03)			0.05 (0.01-0.36)
BMI category	Normal	12 (25.00)	16 (18.39)	1.04	0.594	Reference
	Overweight	24 (50.00)	44 (50.57)			0.73 (0.30-1.79)
	Obese	12 (25.00)	27 (31.03)			0.59 (0.22-1.63)
STEMI location	Lateral + Inferior MI	14 (29.17)	80 (91.95)	57.67	<0.001*	Reference
	Anterior wall MI	34 (70.83)	7 (8.05)			27.76 (10.29-74.85)
Reperfusion strategy	Primary PCI	23 (47.92)	57 (65.52)	5.14	0.076	Reference
	Thrombolytic therapy	16 (33.33)	15 (17.24)			2.64 (1.12-6.21)
	Medical therapy only	9 (18.75)	15 (17.24)			1.49 (0.57-3.87)
Symptom-to-door time	Mean ± SD (hours)	5.77 ± 3.54	3.54 ± 1.83	3059†	<0.001*	1.49 (1.23-1.80)
Total ischemic time	Mean ± SD (hours)	7.38 ± 4.38	4.99 ± 1.89	2790†	0.001*	1.32 (1.14-1.53)

The associations between demographic and clinical characteristics and in-hospital mortality are presented in Table 3. In the multivariable logistic regression analysis, neither gender, age group, BMI category, reperfusion strategy, nor total ischemic time demonstrated statistically significant associations with in-hospital mortality (p > 0.05). Although older age (41-80 years) showed higher crude odds of mortality than younger age (18-40 years), this association was not retained after adjustment. Diabetes mellitus and hypertension emerged as the strongest independent predictors of in-hospital mortality. Patients with diabetes had significantly higher odds of death (adjusted OR = 37.67, 95% CI: 4.94-287.29; p = 0.001), while hypertension was associated with an even greater increase in mortality risk (adjusted OR = 170.50, 95% CI: 5.72-5081.40; p = 0.003). Smoking status showed a significant inverse association in the univariate analysis but was not independently associated with mortality after adjustment (adjusted OR = 0.19, 95% CI: 0.01-2.96; p = 0.234), suggesting that the crude association was likely influenced by confounding. Among infarct characteristics, anterior wall STEMI remained a strong independent predictor of in-hospital mortality compared with lateral and inferior STEMI (adjusted OR = 27.06, 95% CI: 5.17-141.74; p < 0.001). In addition, each one-hour increase in symptom-to-door time was associated with an 89% increase in the odds of in-hospital mortality (adjusted OR = 1.89, 95% CI: 1.19-2.99; p = 0.007). These findings showed that diabetes mellitus, hypertension, anterior wall STEMI, and delayed hospital presentation were the principal independent determinants of in-hospital mortality in this cohort.

**Table 3:** Association of Demographic and Clinical Risk Factors With In-Hospital Mortality Among STEMI Patients *p < 0.05 indicates statistical significance. OR: odds ratio; CI: confidence interval; BMI: body mass index; STEMI: ST-elevation myocardial infarction; PCI: percutaneous coronary intervention

Variable	Category / Comparison	Died n (%)	Survived n (%)	χ²	Crude OR (95% CI)	Adjusted OR (95% CI)	p-value
Gender	Female	Reference	Reference	-	Reference	Reference	-
	Male (vs Female)	21 (43.8)	47 (54.0)	1.31	1.51 (0.75-3.03)	1.69 (0.33-8.56)	0.527
Age group (years)	18-40	Reference	Reference	-	Reference	Reference	-
	41-80 (vs 18-40)	45 (93.8)	72 (82.8)	3.23	3.13 (0.87-11.24)	1.81 (0.13-25.17)	0.658
Diabetes mellitus	No	Reference	Reference	-	Reference	Reference	-
	Yes (vs No)	45 (93.8)	36 (41.4)	35.35	21.25 (6.18-73.05)	37.67 (4.94-287.29)	0.001*
Hypertension	No	Reference	Reference	-	Reference	Reference	-
	Yes (vs No)	47 (97.9)	47 (54.0)	28.18	40.00 (5.35-299.07)	170.50 (5.72-5081.40)	0.003*
Smoking status	Non-smoker	Reference	Reference	-	Reference	Reference	-
	Smoker (vs Non-smoker)	1 (2.1)	27 (31.0)	15.78	0.05 (0.01-0.35)	0.19 (0.01-2.96)	0.234
BMI category	Normal	Reference	Reference	-	Reference	Reference	-
	Overweight (vs Normal)	24 (50.0)	44 (50.6)	1.04	0.73 (0.30-1.77)	0.48 (0.06-3.90)	0.489
	Obese (vs Normal)	12 (25.0)	27 (31.0)	-	0.59 (0.21-1.68)	1.78 (0.15-21.52)	0.649
STEMI location	Lateral + Inferior MI	Reference	Reference	-	Reference	Reference	-
	Anterior wall MI (vs Others)	34 (70.8)	7 (8.0)	57.67	27.76 (10.72-71.89)	27.06 (5.17-141.74)	<0.001*
Reperfusion strategy	Primary PCI	Reference	Reference	-	Reference	Reference	-
	Thrombolytic therapy (vs PCI)	-	-	-	2.64 (1.12-6.21)	5.97 (0.88-40.42)	0.067
	Medical therapy only (vs PCI)	-	-	-	1.49 (0.57-3.87)	2.06 (0.31-13.73)	0.456
Symptom-to-door time	Per hour increase	-	-	-	1.49 (1.23-1.80)	1.89 (1.19-2.99)	0.007*
Total ischemic time	Per hour increase	-	-	-	1.32 (1.14-1.53)	1.18 (0.89-1.57)	0.249

## Discussion

The in-hospital mortality rate among patients with acute STEMI complicated by CS was 35.56% (48/135) in the present study. Although this mortality is lower than that reported in several large international registries, it remains substantial and underscores the severe prognosis associated with CS despite contemporary management. This finding is consistent with the Malaysian NCVD registry, which reported an in-hospital mortality of 34.1% among STEMI patients with CS, while large European and North American registries have reported mortality ranging from 40% to 60%, depending on patient characteristics, reperfusion strategies, and availability of advanced mechanical circulatory support [13-16]. Similarly, the recently published randomized trial demonstrated that CS continues to carry considerable short-term mortality despite advances in revascularization and intensive care management, emphasizing the persistent clinical challenge of this condition [17]. The relatively lower mortality observed in our cohort may reflect differences in patient selection, healthcare resources, and treatment practices compared with high-income healthcare systems.

The mean age in our cohort was 55.03 ± 13.06 years, and patients aged 41-80 years demonstrated a higher mortality rate than those aged 18-40 years (38.46% vs. 16.67%), although this difference did not reach statistical significance (p = 0.072). This finding suggests a trend toward poorer outcomes among older patients and is biologically plausible because increasing age is associated with reduced physiological reserve, greater frailty, more extensive coronary artery disease, and a higher burden of cardiovascular comorbidities. Aijaz et al. [18] found in a large registry study of more than 30,000 ACS patients that increasing age was independently associated with higher mortality, largely owing to impaired compensatory mechanisms, more severe left ventricular dysfunction, and increased susceptibility to multiorgan failure following CS [19]. Although age was not an independent predictor in our unadjusted analysis, the observed trend is consistent with the contemporary literature and may have reached statistical significance in a larger cohort.

Diabetes mellitus was present in 60.0% of patients and showed a strong association with in-hospital mortality (55.56% vs. 5.56%, p < 0.001). Patients with diabetes frequently have diffuse coronary artery disease, endothelial dysfunction, impaired microvascular perfusion, and delayed myocardial recovery following infarction, all of which may contribute to adverse outcomes after CS. Similar findings have been reported by the Malaysian NCVD registry and the Cardiogenic Shock Working Group (CSWG), both of which identified diabetes mellitus as an important predictor of mortality in patients with STEMI complicated by CS despite revascularization and contemporary intensive care management [13,20]. The results highlight the importance of metabolic comorbidities in identifying patients at particularly high risk of adverse in-hospital outcomes.

Hypertension was present in 69.63% of patients and was strongly associated with mortality (50.0% vs. 2.44%, p < 0.001). Chronic hypertension promotes left ventricular hypertrophy, myocardial fibrosis, impaired diastolic filling, and reduced cardiac reserve, thereby limiting the heart's ability to compensate during acute ischemic injury and CS. Similar observations have been reported in large registry studies and contemporary European Society of Cardiology (ESC) guidance, where hypertension has consistently been associated with worse clinical outcomes because of advanced structural heart disease and greater cardiovascular risk burden [16,20-24].

Unexpectedly, smoking status demonstrated an inverse association with in-hospital mortality, with only one death observed among smokers compared with 47 deaths among non-smokers (p < 0.001). This finding should be interpreted with considerable caution and should not be considered evidence of a protective effect of smoking. Rather, it is likely explained by residual confounding, selection bias, the relatively small number of smokers included in the study, and the absence of multivariable adjustment. This phenomenon has previously been described as the "smoker's paradox," whereby smokers presenting with AMI are often younger, have fewer chronic comorbidities, and receive earlier reperfusion therapy than non-smokers. However, large adjusted registry analyses have consistently demonstrated that smoking remains an independent predictor of adverse cardiovascular outcomes after accounting for age, comorbidities, and treatment differences [5,9,25]. Therefore, our findings should be regarded as exploratory rather than causal.

Infarct location had a marked influence on clinical outcomes, with anterior wall STEMI demonstrating the highest in-hospital mortality (82.93%, p < 0.001). Anterior wall STEMI most commonly results from occlusion of the left anterior descending (LAD) coronary artery, leading to infarction of a large proportion of the left ventricular myocardium. Consequently, these patients experience more extensive myocardial necrosis, greater impairment of left ventricular systolic function, reduced cardiac output, and an increased risk of persistent CS and multiorgan hypoperfusion. Evidence indicates that infarct size and the severity of left ventricular dysfunction are among the strongest determinants of mortality in AMI-related CS [20,24,26].

A systematic review and meta-analysis by Maimaitiming et al. identified extensive myocardial injury and reduced left ventricular function as major predictors of both CS and mortality following AMI [11]. Similarly, Hayıroğlu et al. demonstrated that anterior STEMI was independently associated with higher in-hospital mortality among patients with STEMI complicated by CS [10]. Findings from the Bremen STEMI Registry further showed that patients with anterior infarction experienced worse clinical outcomes because of larger infarct burden and more severe hemodynamic compromise [9]. Contemporary reviews and international guidelines likewise recognize extensive anterior infarction as a high-risk clinical phenotype requiring rapid reperfusion and aggressive hemodynamic support owing to its strong association with adverse outcomes [7,14,15,24,26]. Therefore, our findings reinforce that anterior wall STEMI is an important marker of increased in-hospital mortality in patients with STEMI complicated by CS and are consistent with the current understanding that larger infarct size and greater left ventricular systolic dysfunction are principal drivers of poor prognosis in this population.

Strengths

This study has several strengths. First, it employed a prospective observational design with consecutive enrollment of eligible patients, thereby minimizing selection bias and reflecting real-world clinical practice. Second, standardized eligibility criteria were used to identify patients with CS secondary to acute STEMI, ensuring a clinically homogeneous study population. Third, the study provides valuable contemporary data on in-hospital mortality and its associated clinical characteristics from a tertiary-care referral center in Pakistan, where published evidence on CS complicating STEMI remains limited. Fourth, we performed multivariate logistic regression analysis with backward elimination to identify independent predictors of in-hospital mortality, controlling for confounding by reperfusion strategy, time-to-treatment variables, and clinical comorbidities (Table 3). Fifth, critical treatment-related variables - including reperfusion modality, symptom-to-door time, total ischemic time, door-to-balloon time, and door-to-needle time - were systematically collected and incorporated into the multivariate model, strengthening the causal inference of our findings.

Limitations

Several limitations should be acknowledged. This was a single-center study with a relatively small sample size (n = 135), which may limit the generalizability of the findings to other healthcare settings. The outcome assessment was restricted to in-hospital mortality; therefore, longer-term outcomes after hospital discharge could not be evaluated. Left ventricular ejection fraction and detailed angiographic characteristics (e.g., SYNTAX score, culprit vessel patency, multivessel disease) were not systematically documented in the medical records during the study period and therefore could not be incorporated into the analysis. The limited number of mortality events (n = 48) relative to the number of candidate predictors necessitated careful variable selection; we followed the guideline of at least 10 events per predictor variable to avoid overfitting, and the final multivariate model included seven predictors with acceptable calibration (Hosmer-Lemeshow p = 0.725). Reperfusion strategy and time-to-treatment variables were retrospectively retrieved from existing hospital records. While every effort was made to ensure accuracy, some data points may have been incomplete or subject to documentation bias. Despite multivariate adjustment, residual confounding from unmeasured variables (e.g., pre-hospital delays, socioeconomic factors, physician-level treatment preferences, adjunctive pharmacotherapy) may still influence the observed associations. Finally, as with all observational studies, causal relationships between the evaluated clinical characteristics and mortality cannot be established.

## Conclusions

Despite improvements in modern cardiac care, CS after acute STEMI was found to be a severe and fatal complication in this study, with a high in-hospital mortality rate. Key clinical characteristics such as diabetes mellitus, hypertension, smoking status, and anterior wall AMI were significantly associated with mortality, suggesting that metabolic comorbidities and infarct characteristics also have a significant influence on patient mortality. The results highlight the value of early risk stratification, timely reperfusion therapy, and intensive in-hospital care by multiple specialties in the hope of improving outcomes in this high-risk patient population.

## Appendices

Appendix 1. Structured cardiogenic shock - STEMI (CS-STEMI) questionnaire

Section A: Patient Identification

Serial Number: __________________________

Name: _________________________________

Contact Number: ________________________

Address: _______________________________

Hospital Registration/MRN: __________________________

Date of Admission: ____ / ____ / ________

Date of Discharge/Death: ____ / ____ / ________

Section B: Demographic Data

Age (years): ___________________________

Gender: ☐ Male ☐ Female

Marital Status: ☐ Single ☐ Married ☐ Divorced ☐ Widowed

Education Level: ☐ No formal education ☐ Primary ☐ Secondary ☐ Higher secondary ☐ Graduate/Postgraduate

Occupation: ___________________________

Socioeconomic Status: ☐ Low ☐ Middle ☐ High

Section C: Anthropometric Data

Weight (kg): ___________________________

Height (m): ___________________________

Body Mass Index (BMI): _________________

BMI Category: ☐ Normal weight (18.5-24.9 kg/m²) ☐ Overweight (25.0-29.9 kg/m²) ☐ Obese (≥30.0 kg/m²)

Waist Circumference (cm): ___________________________

Hip Circumference (cm): ___________________________

Waist-to-Hip Ratio: ___________________________

Section D: Clinical History

D1. Diabetes Mellitus

☐ Yes ☐ No

If Yes: ☐ Type 1 ☐ Type 2 Duration (years): ___________________________ Treatment: ☐ Diet control only ☐ Oral hypoglycemics ☐ Insulin ☐ Combination Most Recent HbA1c (%): ___________________________ Fasting Blood Glucose on Admission (mg/dL): ___________________________ Random Blood Glucose on Admission (mg/dL): ___________________________

D2. Hypertension

☐ Yes ☐ No

If Yes: Duration (years): ___________________________ Treatment: ☐ Lifestyle modification only ☐ Single agent ☐ Dual therapy ☐ Triple therapy Blood Pressure on Admission: Systolic: __________ mmHg Diastolic: __________ mmHg Mean Arterial Pressure: __________ mmHg

D3. Dyslipidemia

☐ Yes ☐ No

If Yes: ☐ Elevated total cholesterol ☐ Elevated LDL ☐ Low HDL ☐ Elevated triglycerides Most Recent Lipid Profile: Total Cholesterol: __________ mg/dL LDL Cholesterol: __________ mg/dL HDL Cholesterol: __________ mg/dL Triglycerides: __________ mg/dL

D4. Smoking Status

☐ Current Smoker ☐ Former Smoker ☐ Never Smoker

If Current/Former: Pack-Years: ___________________________ Type: ☐ Cigarettes ☐ Hookah/Water pipe ☐ Bidis ☐ Other: __________ Duration (years): ___________________________

D5. Family History of Premature CAD

☐ Yes ☐ No

If Yes, specify relationship: ___________________________

D6. Prior Cardiovascular History

☐ Prior MI ☐ Prior PCI ☐ Prior CABG ☐ Prior Stroke/TIA ☐ Peripheral Artery Disease ☐ Chronic Kidney Disease ☐ Chronic Obstructive Pulmonary Disease ☐ Heart Failure

D7. Prior Medications

☐ Aspirin ☐ Clopidogrel ☐ Statin ☐ Beta-blocker ☐ ACE inhibitor/ARB ☐ Diuretic ☐ Calcium channel blocker ☐ Oral anticoagulant ☐ None

Section E: Index Cardiac Event

E1. Symptom Onset

Date: ____ / ____ / ________ Time: : (AM/PM)

E2. First Medical Contact

Date: ____ / ____ / ________ Time: : (AM/PM) ☐ Self-presented to ED ☐ Referred from another hospital ☐ Brought by ambulance ☐ Other: __________

E3. Hospital Arrival

Date: ____ / ____ / ________ Time: : (AM/PM)

Symptom-to-Door Time (hours): ___________________________

E4. Type of STEMI

☐ Anterior wall MI (V1-V4, I, aVL) ☐ Lateral wall MI (I, aVL, V5-V6) ☐ Inferior wall MI (II, III, aVF) ☐ Posterior wall MI (V7-V9, tall R in V1-V2) ☐ Right ventricular MI (V4R) ☐ Multivessel involvement

ECG Date/Time: ____ / ____ / ________ :

Peak Troponin I/T (ng/mL): ___________________________ Peak CK-MB (U/L): ___________________________

E5. Presence of Cardiogenic Shock

☐ Yes (SBP <90 mmHg for >30 min OR requiring vasopressors/inotropes to maintain SBP ≥90 mmHg, with evidence of tissue hypoperfusion: cold/clammy skin, altered mental status, oliguria <30 mL/h, or serum lactate >2 mmol/L)

Time of Shock Onset: ____ / ____ / ________ :

Shock Duration (hours): ___________________________

Hemodynamic Parameters at Shock Onset: SBP: __________ mmHg DBP: __________ mmHg Heart Rate: __________ bpm Cardiac Index (if available): __________ L/min/m² Pulmonary Capillary Wedge Pressure (if available): __________ mmHg

Section F: Reperfusion and Time Parameters

F1. Reperfusion Strategy

☐ Primary PCI ☐ Rescue PCI (after failed thrombolysis) ☐ Thrombolysis (pharmacologic) ☐ Conservative/Medical management only (no reperfusion attempted)

Decision Time: ____ / ____ / ________ :

F2. For Primary PCI Patients

Door-to-Balloon Time (minutes): ___________________________ Arterial Access: ☐ Radial ☐ Femoral Infarct-Related Artery: ☐ LAD ☐ LCx ☐ RCA ☐ LM ☐ SVG Pre-PCI TIMI Flow: ☐ 0 ☐ 1 ☐ 2 ☐ 3 Post-PCI TIMI Flow: ☐ 0 ☐ 1 ☐ 2 ☐ 3 Stent Deployed: ☐ Yes ☐ No ☐ DES ☐ BMS Glycoprotein IIb/IIIa Inhibitor Used: ☐ Yes ☐ No

F3. For Thrombolysis Patients

Thrombolytic Agent: ☐ Streptokinase ☐ Tenecteplase ☐ Reteplase ☐ Alteplase Door-to-Needle Time (minutes): ___________________________ Rescue PCI Required: ☐ Yes ☐ No

F4. For No Reperfusion Patients

Reason for No Reperfusion: ☐ Late presentation (>12 hours) ☐ Contraindication to thrombolysis ☐ No cath lab availability ☐ Patient/family refusal ☐ Severe comorbidity precluding intervention ☐ Other: __________

F5. Total Ischemic Time

Symptom-to-Reperfusion Time (hours): ___________________________ (For no reperfusion: Symptom-to-Last Assessment Time)

Section G: Management Details

G1. Antiplatelet Therapy

☐ Aspirin (loading dose: ______ mg, maintenance: ______ mg) ☐ Clopidogrel (loading dose: ______ mg, maintenance: ______ mg) ☐ Ticagrelor (loading dose: ______ mg, maintenance: ______ mg) ☐ Prasugrel (loading dose: ______ mg, maintenance: ______ mg)

G2. Anticoagulation

☐ Unfractionated heparin ☐ Enoxaparin ☐ Bivalirudin Dose/Duration: ___________________________

G3. Inotropic/Vasopressor Support

☐ Yes ☐ No

If Yes: ☐ Dopamine ☐ Dobutamine ☐ Norepinephrine ☐ Epinephrine ☐ Vasopressin ☐ Milrinone Maximum Dose: ___________________________ Duration (hours): ___________________________

G4. Mechanical Circulatory Support

☐ Intra-aortic Balloon Pump (IABP) ☐ Impella ☐ Extracorporeal Membrane Oxygenation (ECMO) ☐ None

G5. Ventilatory Support

☐ Non-invasive ventilation (BiPAP/CPAP) ☐ Invasive mechanical ventilation ☐ None

G6. Renal Replacement Therapy

☐ Yes ☐ No

G7. ICU/CCU Admission

☐ Yes ☐ No

ICU/CCU Length of Stay (days): ___________________________

Section H: Echocardiographic Data

Date of Echocardiogram: ____ / ____ / ________

Left Ventricular Ejection Fraction (%): ___________________________

Left Ventricular End-Diastolic Diameter (mm): ___________________________

Left Ventricular End-Systolic Diameter (mm): ___________________________

Regional Wall Motion Abnormality: ☐ Anterior ☐ Inferior ☐ Lateral ☐ Septal ☐ Apical

Mitral Regurgitation: ☐ None ☐ Mild ☐ Moderate ☐ Severe

Right Ventricular Function: ☐ Normal ☐ Mildly reduced ☐ Moderately reduced ☐ Severely reduced

Section I: Laboratory Data on Admission

Hemoglobin (g/dL): ___________________________

White Blood Cell Count (×10³/μL): ___________________________

Platelet Count (×10³/μL): ___________________________

Serum Creatinine (mg/dL): ___________________________

Blood Urea Nitrogen (mg/dL): ___________________________

eGFR (mL/min/1.73m²): ___________________________

Serum Sodium (mEq/L): ___________________________

Serum Potassium (mEq/L): ___________________________

Serum Lactate (mmol/L): ___________________________

Arterial Blood Gas pH: ___________________________

PaO₂ (mmHg): ___________________________

PaCO₂ (mmHg): ___________________________

HCO₃⁻ (mEq/L): ___________________________

Section J: Outcome

J1. In-Hospital Outcome

☐ Survived to discharge ☐ Died

If Died: Date/Time of Death: ____ / ____ / ________ :

Cause of Death: ☐ Refractory cardiogenic shock ☐ Fatal arrhythmia (VT/VF/asystole/PEA) ☐ Mechanical complication (free wall rupture/VSD/papillary muscle rupture) ☐ Stroke ☐ Multi-organ failure ☐ Sepsis ☐ Other: __________

J2. Duration of Hospital Stay

Total (days): ___________________________

ICU/CCU (days): ___________________________

Ward (days): ___________________________

J3. Discharge Medications (for survivors)

☐ Aspirin ☐ Clopidogrel/Ticagrelor/Prasugrel ☐ Statin ☐ Beta-blocker ☐ ACE inhibitor/ARB ☐ Aldosterone antagonist ☐ Diuretic ☐ Oral anticoagulant

Section K: Data Collector Verification

Data Collector Name: ___________________________

Signature: ___________________________

Date: ____ / ____ / ________

Supervisor Review: ☐ Verified ☐ Queries Raised

Supervisor Name: ___________________________

Signature: ___________________________

Date: ____ / ____ / ________
